# Choroidal Remodeling After Subthreshold Micropulse Laser in Chronic Central Serous Chorioretinopathy: Short-Term Outcomes

**DOI:** 10.3390/jcm14020306

**Published:** 2025-01-07

**Authors:** Pasquale Viggiano, Giacomo Scotti, Alba Chiara Termite, Alfonso Savastano, Giacomo Boscia, Arcangelo Clemente, Antonio Salvelli, Ermete Giancipoli, Francesco Pignatelli, Federica Evangelista, Giovanni Alessio, Francesco Boscia

**Affiliations:** 1 Department of Translational Biomedicine Neuroscience, University of Bari “Aldo Moro”, 70121 Bari, Italy; giacomo.scotti@tiscali.it (G.S.); a.termite2@studenti.uniba.it (A.C.T.); bosciagiacomo@gmail.com (G.B.); a.clemente39@studenti.uniba.it (A.C.); asavastano21@gmail.com (A.S.); giovanni.alessio@uniba.it (G.A.); francescoboscia@hotmail.com (F.B.); 2 Department of Ophthalmology, Ente Ecclesiastico Ospedale Generale Regionale “F. Miulli”, 70021 Acquaviva delle Fonti, Italy; a.salvelli@studenti.uniba.it (A.S.); f.evangelista@miulli.it (F.E.); 3 Ophthalmology Unit, Libera Università Mediterranea Degennaro, 70010 Casamassima, Italy; 4 Department of Ophthalmology, University of Foggia, 71122 Foggia, Italy; ermete.giancipoli@unifg.it; 5 Eye Clinic, “SS. Annunziata” Hospital, ASL Taranto, 74121 Taranto, Italy; pignatelli.oculista@gmail.com

**Keywords:** OCT, choroid, micropulse laser treatment

## Abstract

**Objectives**: To evaluate the effects of subthreshold micropulse laser treatment (SMLT) on choroidal architecture in chronic central serous chorioretinopathy (CSC) and their correlations with functional outcomes. **Methods**: This retrospective study included 48 eyes with chronic CSC that were treated with 577 nm SMLT. The choroidal thickness (CT); Sattler’s layer and choriocapillaris complex thickness (SLCCT); Haller’s layer thickness (HLT); subretinal fluid (SRF); and best-corrected visual acuity (BCVA) were assessed at baseline and at 2 months post treatment. **Results**: At 2 months, the SLCCT increased from 185.92 ± 80.89 μm to 214.17 ± 83.36 μm (*p* = 0.023), and the total CT increased from 444.46 ± 80.43 μm to 484.33 ± 93.19 μm (*p* = 0.002). The SRF height decreased from 140.38 ± 95.89 μm to 57.58 ± 63.54 μm (*p* < 0.001), with complete resolution in 79.2% of cases. The BCVA improved from 0.41 ± 0.48 to 0.22 ± 0.30 logMAR (*p* < 0.001). Changes in the SLCCT correlated negatively with BCVA changes (r = −0.48, *p* = 0.025) and positively with total CT changes (r = 0.687, *p* < 0.001). **Conclusions**: SMLT induces significant choroidal remodeling in chronic CSC, particularly affecting the Sattler–Bruch layer complex. The increase in the SLCCT correlates with visual improvement, challenging the conventional understanding of choroidal thinning in CSC treatment.

## 1. Introduction

Central serous chorioretinopathy (CSC) is a chorioretinal disorder characterized by the serous detachment of the neurosensory retina, often associated with the focal detachment of the retinal pigment epithelium (RPE) [1,2]. This condition primarily affects young to middle-aged adults and is more prevalent in men [3]. While the exact pathophysiology of CSC remains incompletely understood, choroidal vascular hyperpermeability and dysfunction are believed to play crucial roles in its development and persistence [3,4].

The natural course of CSC is typically self-limiting, with spontaneous resolution occurring in many acute cases within 3–4 months. However, a significant proportion of patients experience chronic or recurrent episodes, leading to progressive visual impairment and potential permanent structural changes in the retina and choroid [5]. These cases pose a therapeutic challenge and underscore the need for effective interventions.

Various treatment modalities have been employed in the management of chronic CSC (cCSC), including observation, photodynamic therapy (PDT), conventional laser photocoagulation, and, more recently, subthreshold micropulse laser treatment (SMLT) [6]. Among these, SMLT has gained increasing attention due to its potential to achieve therapeutic effects while minimizing collateral thermal damage to the retina and choroid [7].

SMLT delivers a series of ultra-short laser pulses, allowing the tissue to cool between pulses and, theoretically, reducing the risk of thermal injury [8]. This approach is thought to stimulate the RPE and modulate choroidal vascular permeability without causing visible laser burns. However, the precise mechanisms by which SMLT affects the choroidal architecture and facilitates the resolution of subretinal fluid in CSC remain to be fully elucidated.

Recent advancements in retinal imaging, particularly optical coherence tomography (OCT) and enhanced depth imaging OCT (EDI-OCT), have significantly improved our ability to visualize and quantify choroidal structures [9]. These technologies enable the detailed examination of choroidal layers, including the Haller’s layer (large choroidal vessels) and the Sattler’s layer (medium choroidal vessels), as well as the accurate measurement of subretinal fluid (SRF) and choroidal thickness (CT) [10].

Despite these technological advancements and the growing use of SMLT, there is a paucity of data on the specific effects of this treatment on individual choroidal layers in CSC. Furthermore, the relationship between changes in choroidal architecture and the resolution of SRF following SMLT remains poorly understood.

In this study, we aim to address this knowledge gap by investigating the changes in choroidal structure, particularly focusing on the Haller’s and Sattler’s layers, and their correlation with SRF reduction in patients with cCSC treated with SMLT. By analyzing these parameters before and 2 months after treatment, we seek to provide insights into the potential mechanisms of action of SMLT and its effects on choroidal remodeling in cCSC.

## 2. Materials and Methods

### 2.1. Study Participants

This retrospective study was conducted at the medical retina clinic of the Department of Translational Biomedicine Neuroscience at the Aldo Moro University in Bari, Italy. We included 48 patients diagnosed with chronic CSC who underwent 577 nm SMLT using the Navilas^®^ 577s system (Navilas GmbH, Teltow, Germany) between January 2023 and January 2024. The study was conducted in accordance with the tenets of the Declaration of Helsinki. As per Italian regulations for retrospective studies, formal approval from an ethics committee was not required, but the institutional ethics committee was informed about the study.

The inclusion criteria were as follows: (1) age ≥ 18 years; (2) a diagnosis of chronic CSC, confirmed by fluorescein angiography (FA), indocyanine green angiography (ICGA), and optical coherence tomography (OCT); (3) the presence of subretinal fluid for at least 3 months; (4) a best-corrected visual acuity (BCVA) of 20/400 or better; (5) no previous treatments for CSC, with SMLT representing the first intervention; and (6) only one eye per patient was included in the study, even if both eyes met the inclusion criteria. In cases where both eyes were eligible, the eye with the longer duration of symptoms was selected. Patients were excluded from the study if they had undergone previous ophthalmological surgery, laser therapy, or PDT. Additionally, those with a history of anti-VEGF treatment, the presence of MNV assessed with OCT angiography (OCTA) and ICGA, systemic steroid use, other retinal disorders, pregnancy, and media opacities preventing high-quality imaging were not included in the study.

Patients were treated after a minimum of 3 months of persistent SRF, with the treatment being initiated at the time of the diagnosis of chronicity, rather than waiting for the development of extensive RPE changes.

### 2.2. Examinations

All the patients underwent comprehensive ophthalmic examinations at baseline and 2 months after treatment. These examinations included BCVA measurements using Early Treatment Diabetic Retinopathy Study (ETDRS) charts, slit-lamp biomicroscopy, dilated fundus examination, and OCT imaging. FA and ICGA were performed at baseline for diagnostic purposes. FAG was used to confirm the diagnosis of cCSC and identify the presence and location of leakage points. ICGA was employed to evaluate choroidal hyperpermeability (CH).

### 2.3. OCT Imaging Analysis

**Figure 1:**
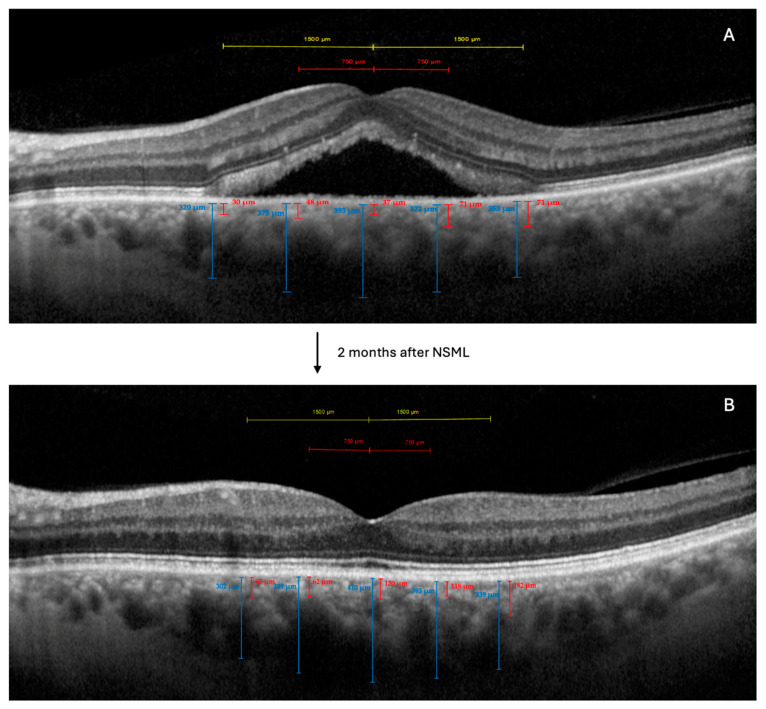
Enhanced-depth imaging optical coherence tomography (EDI-OCT) images of a patient with chronic central serous chorioretinopathy (cCSC), before and after navigated subthreshold micropulse laser treatment (NSML). (**A**): Baseline EDI-OCT image showing subretinal fluid (SRF) and choroidal thickness measurements at five locations: subfoveal, and at 750 μm and 1500 μm nasal and temporal to the fovea. (**B**): EDI-OCT image 2 months after SMLT, demonstrating resolution of SRF and changes in choroidal thickness. Note the increase in Sattler’s layer and choriocapillaris complex thickness (SLCCT) and total choroidal thickness (CT) compared to baseline. Vertical lines indicate choroidal thickness measurements. The upper portion of each line represents the outer border of the retinal pigment epithelium, while the lower end marks the choroidal–scleral interface.

OCT imaging was performed using Spectralis ^®^ HRA+OCT (Heidelberg Engineering; Heidelberg, Germany) with the enhanced depth imaging (EDI) mode. The OCT images of the macular area were obtained with 49 horizontal, raster-dense, linear B-scans, centered on the fovea (Figure 1). At baseline, we selected the horizontal high-resolution EDI-OCT scan that showed the maximum SRF. For the follow-up visits, we used the instrument’s reference function to ensure that the same retinal location was scanned, allowing for a precise comparison with the baseline measurements (Figure 1).

The choroidal thickness measurements were performed manually, using the caliper function of the OCT software (Heyex 2) [10]. We measured the following parameters:

1. Total choroidal thickness (CT): defined as the distance from the outer border of the retinal pigment epithelium to the choroidal–scleral interface.
2. Haller’s layer thickness (HLT): defined as the distance from the choroidal–scleral interface to the inner border of the large choroidal vessel layer [10].
3. Sattler’s layer and choriocapillaris complex thickness (SLCCT): calculated as the difference between the CT and the HLT [10].

These measurements were taken at five locations: subfoveal, and at 750 μm and 1500 μm nasal and temporal to the fovea. The average of these five measurements was used for the analysis [10].

The subretinal hyper-reflective material (SHRM) thickness was measured using the method described by Sadda et al. [11]. The SHRM was identified in the subretinal space and measured from the inner surface of the hyper-reflective material to the inner surface of the RPE (if present) or to the Bruch’s membrane if the RPE was absent.

The SRF height was measured as the maximum vertical distance between the outer border of the neurosensory retina and the retinal pigment epithelium.

The presence of pigment epithelial detachment (DEP) was also evaluated and recorded as present or absent.

All the measurements were performed by two experienced graders (PV and GB), independently. In the case of discrepancies greater than 15% between the two graders, a third grader (FB) adjudicated the measurement. The average of the two closest measurements was used for the analysis.

### 2.4. Outcome Measures

This study evaluated several key outcomes from baseline to 2 months after treatment. We assessed changes in SLCCT, CT, and HLT. Additionally, we measured alterations in the SHRM thickness and the SRF height. The presence or absence of DEP was also noted. Finally, we evaluated changes in the BCVA to assess the functional impact of the treatment.

### 2.5. 577 nm Subthreshold Micropulse Laser Treatment

SMLT was performed using the Navilas^®^ 577s system. After topical anesthesia, the laser was applied exclusively to the area of subretinal fluid (SRF), starting from the subfoveal region. The laser parameters were as follows: 200 μm spot size, 0.2 s duration, 5% duty cycle, and the power titrated to be just below the visible threshold burn. Multiple confluent spots were applied to cover the entire area of SRF. These specific treatment parameters were consistently used for all the patients in the study sample.

### 2.6. Statistical Analysis

The statistical analysis was performed using SPSS version 20.0 (SPSS Inc., Chicago, IL, USA). Continuous variables were expressed as the mean ± standard deviation. Paired *t*-tests were used to compare the pre- and post-treatment measurements for the continuous variables, such as the SLCCT, CT, HLT, SHRM thickness, and SRF height. For the categorical variable of DEP presence, McNemar’s test was used to assess the changes from baseline to post treatment. To evaluate the relationship between anatomical changes and visual outcomes, we performed correlation analyses between changes in the OCT parameters (SLCCT, CT, HLT, SHRM thickness, and SRF height) and changes in the BCVA. Pearson’s correlation coefficient was used for these analyses. A *p*-value of <0.05 was considered statistically significant for all the analyses. To account for multiple comparisons, we applied the Bonferroni correction where appropriate.

## 3. Results

### 3.1. Demographic Characteristics

This retrospective study included 48 eyes from 48 patients with cCSC who underwent SMLT. The study population comprised 30 men and 18 women. The mean age of the study participants was 51.0 ± 9.2 years. The mean duration of the CSC symptoms prior to treatment was 6.43 ± 2.1 months. Regarding medical history, 12 patients (25%) had a history of hypertension. Notably, none of the patients reported the current use of steroid medications. All the patients presented with subfoveal SRF and demonstrated choroidal hyperpermeability on ICGA. FA revealed that 40 patients (83%) had a single leakage point, while 8 patients (17%) exhibited multiple leakage points. The mean axial length of the studied eyes was 23.8 ± 1.2 mm. Additionally, 8 patients (16.7%) showed focal RPE atrophy. The characteristics of the subjects included in the analysis are shown in Table 1.

### 3.2. Baseline Measurements and Changes After Treatment

The mean SLCCT increased from 185.92 ± 80.89 μm at baseline to 214.17 ± 83.36 μm at 2 months, representing a mean increase of 28.25 μm (*p* = 0.023). The HLT increased from 258.54 ± 76.57 μm at baseline to 270.17 ± 74.91 μm at 2 months, with a mean increase of 11.63 μm (*p* = 0.223). The total CT increased from 444.46 ± 80.43 μm at baseline to 484.33 ± 93.19 μm at 2 months, showing a mean increase of 39.87 μm (*p* = 0.002). The SRF height showed a marked reduction from a baseline of 140.38 ± 95.89 μm to 57.58 ± 63.54 μm at 2 months, representing a substantial mean decrease of 82.80 μm (*p* < 0.001). Notably, 38 out of the 48 patients (79.2%) showed the complete resolution of the SRF at the 2-month follow-up.

The SHRM thickness decreased from 53.04 ± 48.14 μm at baseline to 32.17 ± 41.42 μm at 2 months, showing a mean reduction of 20.87 μm (*p* = 0.031). Importantly, 40 out of the 48 patients (83.3%) demonstrated the complete resolution of the SHRM at the 2-month follow-up. The presence of DEP remained unchanged from baseline to the 2-month follow-up, with 8 eyes (16.7%) showing DEP at both time points (*p* = 1.000). The BCVA improved significantly from 0.41 ± 0.48 logMAR at baseline to 0.22 ± 0.30 logMAR at 2 months (*p* < 0.001) (Table 2).

### 3.3. Correlations with BCVA

The analysis of correlations revealed several significant associations between anatomical changes and functional outcomes. Changes in the SLCCT showed a moderate negative correlation with changes in the BCVA (r = −0.48, *p* = 0.025), indicating that greater increases in the SLCCT were associated with greater improvements in visual acuity. The reduction in the SHRM thickness demonstrated a moderate positive correlation with the BCVA improvement (r = 0.53, *p* < 0.001), suggesting that the SHRM resolution contributes to visual acuity gains. Additionally, the decrease in the SRF height exhibited a weak-to-moderate correlation with BCVA improvement (r = 0.39, *p* = 0.006) (Table 3).

### 3.4. Correlations Between Anatomical Parameters

Regarding correlations between anatomical parameters, a moderate negative correlation was observed between the change in the SRF and the change in the SLCCT (r = −0.412, *p* = 0.045). A strong positive correlation emerged between the change in the SLCCT and the change in the total CT (r = 0.687, *p* < 0.001), indicating that the increase in the CT is largely driven by the thickening of the SLCCT. The change in the SRF showed a weak positive correlation, although not statistically significant, with the change in the SHRM (r = 0.395, *p* = 0.056) (Table 3).

## 4. Discussion

The present study provides novel insights into the effects of SMLT on choroidal architecture and its relationship with functional outcomes in cCSC patients. The use of SMLT in cCSC has been increasingly studied over the past decade. Scholz et al. [7] conducted a prospective study on 38 eyes with chronic CSC, reporting a significant reduction in SRF and an improvement in BCVA at 6 weeks post treatment. Similarly, Ambiya et al. [12] observed complete SRF resolution in 40% of treated eyes at 4 weeks, with this proportion increasing to 65% at 12 weeks. More recently, a randomized clinical trial by van Dijk et al. [13] compared SMLT with half-dose PDT in chronic CSC. While both treatments were effective, SMLT showed a more favorable safety profile and was associated with fewer adverse events. These findings align with our results, where 79.2% of patients showed complete SRF resolution at 2 months post SMLT.

The mechanism of the action of SMLT in CSC has been attributed to its effects on the RPE and the choroid. Malik et al. [14] proposed that SMLT stimulates the RPE, enhancing its pump function and performing the vectorial transport of fluid. However, the direct effects of SMLT on choroidal architecture, particularly on individual choroidal layers, have not been extensively explored in previous studies. Our study provides a unique perspective on the effects of SMLT on choroidal architecture in CSC. We observed significant increases in the SLCCT and the total CT following SMLT. This finding contrasts with the traditional understanding of CSC treatment outcomes, where a reduction in the CT is often associated with successful therapy [4].

Interestingly, our findings regarding SLCCT changes contrast with some previous reports in the literature. For instance, Torrellas et al. [15] documented a reduction in the Sattler’s layer thickness following laser treatment, which was interpreted as beneficial due to the presumed reduction in choroidal hyperpermeability. Several factors may explain these discrepant findings. First, differences in laser parameters could play a crucial role—our protocol, which used 577 nm SMLT with specific parameters (200 μm spot size, 0.2 s duration, and 5% duty cycle), may induce different tissue responses compared to other protocols. Second, our approach of treating the entire area of the SRF, including the subfoveal region, might result in a different pattern of choroidal response compared to fovea-sparing techniques. Third, the timing of the measurements could be critical—our 2-month follow-up may capture an active remodeling phase that differs from the long-term structural changes. Moreover, the observed SLCCT thickening in our study might represent a compensatory mechanism, reflecting the restoration of normal choroidal autoregulation.

This result suggests that SMLT may induce a remodeling process within the choroid, particularly affecting the Sattler’s layer and the choriocapillaris. This choroidal remodeling could represent a normalization of the choroidal structure and function, rather than simply a reduction in choroidal congestion or hyperpermeability. Interestingly, the increase in the SLCCT showed a moderate negative correlation with changes in the BCVA, indicating that greater increases in the SLCCT were associated with greater improvements in visual acuity. This finding challenges the conventional notion that choroidal thinning is necessary for visual improvement in CSC and suggests a more complex relationship between choroidal structure and visual function.

While our study focused on structural changes in choroidal architecture through EDI-OCT, previous studies, using optical coherence tomography angiography (OCT-A), have provided complementary insights into the microvascular changes following SMLT [16]. Recent research has demonstrated that SMLT can induce significant alterations in choroidal perfusion and CC flow. For instance, studies have shown improvements in CC perfusion following SMLT treatment, suggesting a direct effect on choroidal microvascular function [16]. These vascular changes may help explain the mechanism behind the choroidal remodeling we observed in our study. The increase in the SLCCT that we documented could reflect not only structural reorganization but also functional improvements in choroidal perfusion. This interpretation is supported by previous OCT-A studies, which showed enhanced flow signals in the choriocapillaris layer post SMLT treatment.

The Sattler’s layer, comprised of medium-sized choroidal vessels, and the choriocapillaris, are responsible for the blood supply to the outer retina and the RPE [17,18,19]. In CSC and the broader pachychoroid spectrum disorders, CH and dysfunction are thought to be key pathogenic factors. Importantly, recent research has highlighted the critical role of inner choroidal attenuation, particularly in the Sattler’s layer and choriocapillaris, in the pathogenesis of CSC [18,20,21,22,23]. This attenuation is often accompanied by the dilation of outer choroidal vessels, creating a characteristic pachychoroid appearance [24,25]. The thinning of these inner choroidal layers may compromise their ability to regulate choroidal blood flow and maintain the outer blood–retinal barrier, potentially contributing to the development of CSC [23].

Our results suggest that SMLT may specifically target and modulate the function of these layers, potentially normalizing choroidal blood flow and reducing hyperpermeability. The strong positive correlation observed between changes in the SLCCT and the total CT indicates that, in this specific case of SMLT treatment, the increase in the CT is largely driven by changes in the Sattler–Bruch layer complex. It is important to note that this relationship may not hold true in all cases of CSC or other choroidal pathologies, where changes in the CT can be influenced by various factors and layers.

Moreover, the positive correlation between SLCCT thickening and visual improvement in our CSC cohort raises interesting parallels with other retinal conditions. In high myopia, choroidal thinning is associated with reduced visual acuity [26], while in age-related macular degeneration and diabetic retinopathy [27], choroidal thickness changes can affect visual function [28]. This suggests that an optimal choroidal thickness (rather than simply a reduced thickness) may be important for visual function across various retinal pathologies.

Moreover, the moderate negative correlation between changes in the SRF and the SLCCT suggests a direct relationship between the thickening of the Sattler–Bruch layer complex and the resolution of the SRF. This relationship could be mediated through improved choroidal function and reduced hyperpermeability, ultimately leading to enhanced fluid absorption by the RPE.

The observed thickening of the Sattler–Bruch layer complex following SMLT is an intriguing finding that warrants further investigation. Several hypotheses can be proposed to explain this phenomenon. One possibility is the restoration of vascular autoregulation, where SMLT may restore normal vascular tone in the Sattler’s layer and choriocapillaris, leading to improved blood flow regulation and a more physiological thickness [29]. Multiple mechanisms may contribute to the observed choroidal thickening beyond vascular regulation. These could include the vasodilation of choroidal vessels, enhanced extracellular matrix deposition, and changes in choroidal blood flow dynamics. Rather than being mutually exclusive, these mechanisms might work in concert to achieve the structural and functional normalization we observed. Another plausible explanation relates to enhanced RPE pump function. As suggested by previous studies [30,31], SMLT may stimulate RPE activity. This enhanced RPE function could lead to increased metabolic demands, necessitating an improved blood supply from the Sattler’s layer and the choriocapillaris, which may manifest as the thickening of these layers [32]. Paradoxically, the thickening could also represent a normalization of choroidal structure following the resolution of pathological congestion in other choroidal components. Furthermore, SMLT may induce a controlled, beneficial inflammatory response that promotes tissue repair and remodeling in the choroid. This inflammatory process could contribute to the observed thickening through various cellular and molecular mechanisms. It is important to note that these hypotheses are not mutually exclusive, and the observed thickening may result from a combination of these mechanisms.

The findings of our study have several important clinical implications. First, they suggest that SLCCT could be a valuable biomarker for monitoring treatment response in chronic CSC. The correlation between the SLCCT increase and visual improvement indicates that this parameter may be a useful predictor of functional outcomes. Second, our results challenge the conventional focus on choroidal thinning as a therapeutic goal in CSC. Instead, they suggest that the normalization of the choroidal structure, particularly in the Sattler–Bruch layer complex, may be more important for achieving optimal outcomes. Third, the specific effects of SMLT on the Sattler–Bruch layer complex open new avenues for targeted therapies in CSC. Future treatments could be designed to specifically modulate the function of these choroidal layers, potentially leading to more effective and tailored approaches.

While our study provides novel insights into choroidal remodeling after SMLT, several limitations should be acknowledged. The retrospective nature of our study limits the ability to control for all potential confounding factors and may introduce a selection bias. The lack of a control group limits our ability to definitively attribute the observed changes to SMLT, rather than the natural course of the disease. Similarly, the absence of comparisons with other treatment modalities (such as PDT) prevents the direct assessment of SMLT’s relative efficacy. The 2-month follow-up period, while sufficient to observe initial changes, may not capture the long-term effects or the stability of the observed choroidal remodeling, particularly regarding recurrence rates. The relatively high proportion of women in our cohort (37%) compared to typical CSC populations might reflect regional variations or referral patterns, although the subgroup analysis showed no significant differences in the treatment outcomes between the male and female patients. While our measurements were performed by experienced graders, the manual segmentation of choroidal layers is subject to some degree of inter-observer variability. Our study focused on structural changes but did not include an OCT-A assessment, which could have provided additional insights into the functional implications of the observed choroidal remodeling. The relatively low rate of RPE atrophy (16.7%) in our cohort may reflect our approach of early intervention after establishing chronicity, which could affect the generalizability of our results to more advanced cases. Although we excluded patients with known confounding factors, unidentified systemic or ocular conditions could potentially influence the choroidal thickness and the treatment response.

In conclusion, our study demonstrates that SMLT induces the significant remodeling of the choroidal architecture in CSC, particularly affecting the Sattler–Bruch layer complex. The observed thickening of this complex, coupled with its correlation with visual improvement and SRF resolution, suggests a central role for these choroidal layers in the pathogenesis and treatment response of CSC. These findings challenge the current paradigms in cCSC management and highlight the need for a more nuanced understanding of choroidal structure and function in this condition. Future studies should focus on elucidating the mechanisms underlying these choroidal changes and exploring their long-term implications for cCSC management.

## Figures and Tables

**Table 1:** The clinical and medical characteristics of subjects included in the analysis.

Variables	SMLT Patients (*n* = 48)
Age (years)	51.0 ± 9.27
Gender (female, %)	18 (37%)
Axial length (mm)	23.8 ± 1.2
cCSC duration (months)	6.43 ± 2.1
Hypertension, *n* (%)	12 (25%)
Focal RPE atrophy, *n* (%)	8 (16.7%)
PED, *n* (%)	8 (16.7%)
Single leakage point, *n* (%)	40 (83%)
Multiple leakage points, *n* (%)	8 (17%)
Foveal SRF, *n* (%)	48 (100%)
Steroid use, *n* (%)	0 (0)

Baseline clinical characteristics of patients. cCSC, chronic central serous chorioretinopathy; RPE, retinal pigment epithelium; PED, pigment epithelium detachment; SRF, subretinal fluid.

**Table 2:** Functional and anatomical (OCT) outcomes. Data and comparisons.

	Baseline	2 Months	*p* Value
BCVA (logMAR)	0.41 ± 0.48	0.22 ± 0.30	<0.001
SLCCT (μm)	185.92 ± 80.89	214.17 ± 83.36	0.023
HLT (μm)	258.54 ± 76.57	270.17 ± 74.91	0.223
Total CT (μm)	444.46 ± 80.43	484.33 ± 93.19	0.002
SRF height (μm)	140.38 ± 95.89	57.58 ± 63.54	<0.001
SHRM (μm)	53.04 ± 48.14	32.17 ± 41.42	0.031
DEP, *n*	8	8	1.000

Data are presented as mean ± SD. *p* values represent significance of comparison to baseline data of visual and anatomical parameters at each time point. BCVA, best-corrected visual acuity; SLCCT, Sattler’s layer and choriocapillaris complex thickness; HLT, Haller’s layer thickness; SRF, subretinal fluid CT choroidal thickness; SHRM, subretinal hyper-reflective material; DEP, pigment epithelial detachment.

**Table 3:** Anatomical and functional correlations post SMLT.

Variables		Correlation Coefficient (r)	*p* Value
Δ SLCCT	Δ BCVA (logMAR)	−0.48 *	0.025
Δ HLT	Δ BCVA (logMAR)	0.10	0.178
Δ Total CT	Δ BCVA (logMAR)	0.15	0.163
Δ SHRM	Δ BCVA (logMAR)	0.53 **	<0.001
Δ SRF height	Δ BCVA (logMAR)	0.39	*p* = 0.006
Δ SLCCT	Δ SRF height	−0.41 *	0.045
Δ SLCCT	Δ Total CT	0.68 **	<0.001
Δ SLCCT	Δ HLT	0.21	0.322
Δ SRF height	Δ SHRM	0.39	0.056
Δ SRF height	Δ Total CT	−0.32	0.118
Δ SRF height	Δ HLT	0.10	0.632

Correlations were calculated using Pearson’s correlation coefficient (r). *p*-values are reported for one-tailed tests. * Correlation is statistically significant at *p* < 0.05 level. ** Correlation is statistically significant at *p* < 0.01 level. BCVA, best-corrected visual acuity; SLCCT, Sattler’s layer and choriocapillaris complex thickness; HLT, Haller’s layer thickness; SRF, subretinal fluid; CT, choroidal thickness; SHRM, subretinal hyper-reflective material.

## Data Availability

The data presented in this study are available on request from the corresponding author. The data are not publicly available due to privacy and ethical restrictions, as they contain information that could compromise the privacy of the research participants.
